# Extent of alveolar collapse in expiratory CT as a prognostic marker in idiopathic pulmonary fibrosis

**DOI:** 10.1371/journal.pone.0345308

**Published:** 2026-03-17

**Authors:** Sarah C. Scharm, Cornelia Schaefer-Prokop, Anton Schreuder, Jonathan Ehmig, Anna Hunkemöller, Jan Fuge, Benjamin Seeliger, Jonas Schupp, Frank K. Wacker, Hoen-oh Shin

**Affiliations:** 1 Institute of Diagnostic and Interventional Radiology, Hannover Medical School, Hannover, Germany; 2 Biomedical Research in Endstage and Obstructive Lung Disease Hannover (BREATH), German Center for Lung Research (DZL), Hannover, Germany; 3 Department of Radiology, Radboud University, Nijmegen, The Netherlands; 4 Department of Radiology, Meander Medical Center, Amersfoort, The Netherlands; 5 Leiden Institute of Advanced Computer Science, Leiden University, Leiden, The Netherlands; 6 Dutch Knowledge Centre for Youth Health (NCJ), Utrecht, Netherlands,; 7 Institute of Diagnostic and Interventional Radiology, University Medical Center Göttingen, Göttingen, Germany; 8 Department of Respiratory Medicine and Infectious Diseases, Hannover Medical School, Hannover, Germany; Medical Center - University of Freiburg, GERMANY

## Abstract

To evaluate whether distribution measures of CT-based attenuation histograms in inspiration and expiration can indicate alveolar collapse and serve as a predictive marker in patients with idiopathic pulmonary fibrosis (IPF). This single-center retrospective longitudinal study analyzed CT scans of IPF patients in inspiration and expiration. The patient population was divided into two subgroups based on their status 3 years after baseline CT (death or transplantation versus clinical surveillance). Attenuation histograms in inspiration and expiration were created and analyzed. A Mann-Whitney U test was conducted to assess the difference of CT-derived histogram measures (including skewness) between the two subgroups. Logistic regression was applied to model the ability to distinguish between subgroups using baseline forced vital capacity (FVC%) and CT-derived histogram measures. The study included 66 patients (mean age 69.5 ± 10.9 years, 58 males). After the individual three-year observation period, 37 patients were still alive while 29 had either died or received a transplantation. The two patient subgroups were significantly different in terms of all CT-derived histogram measures and the baseline FVC%. A logistic regression model that only included the CT-derived histogram measure skewness had a better predictive performance (AUC = 0.793, 95% CI = 0.685–0.900) compared to the FVC% model alone (0.708, 0.581–0.836). Whereas further evaluation is needed, paired inspiratory/expiratory attenuation histogram analysis offers a promising approach as a prognostic imaging marker to improve outcome prediction and assess alveolar collapse in IPF.

## Introduction

Computed tomography (CT) scans are routinely performed in patients with idiopathic pulmonary fibrosis (IPF) and play an important role in monitoring disease progression, which varies widely among patients [1,2]. In patients with progressive fibrosis and worsening lung function, antifibrotic medications are recommended to potentially slow down the disease [3,4]. Prospective disease monitoring is desirable and could potentially lead to earlier changes in therapy and improved clinical outcomes for patients.

The visual assessment of extent and progression of disease on CT is cumbersome and prone to interobserver variability [5]. In order to address the limitations of visual assessment, a range of computerized tools have been developed for quantitative CT analysis. The objective of these tools is to obtain more objective and reproducible measurements [6,7]. These include texture-based methods [8] as well as the analysis of density histograms [9]. It was shown in several studies that parameters derived from CTs obtained in inspiration correlate with pulmonary function at the time of imaging [10–12], and some of them were predictive of mortality [13].

In 1999, Günther et al described the concept of alveolar collapse in expiration as the underlying pathophysiological mechanism for development of fibrosis [14]. Subsequent studies identified a disturbance in the protein composition that resulted in increased surface tension of the alveolar walls. This disturbance was shown to be the pathway for alveolar collapse in IPF [15].

Petroulia et al were the first to describe the correlation between alveolar collapse and abnormal density increase comparing inspiration and expiration lung CTs in patients suffering from IPF compared to normal lungs [16]. Similarly, Sul et al found significant differences between healthy lungs and IPF lungs when analyzing attenuation histograms in inspiration and expiration [17]. More recent studies used advanced processing to voxel-wise register inspiratory and expiratory CT scans demonstrating promising results for disease prognostication. Pathologically increased ventilation – defined as pathologically increased density and volume change within the respiratory cycle per voxel – was found to be an indicator of future fibrotic changes [18,19]. The utilization and evaluation of expiratory CT scans in the context of IPF is becoming more widespread, contributing to the development of our understanding of the disease [20,21].

This method, however, requires advanced computer applications that are technically demanding and are not yet broadly applicable.

Aim of this study was therefore to explore whether attenuation histogram measures obtained in paired inspiration/expiration can be used to illustrate the functional concept of alveolar collapse as an abnormally strong attenuation increase. This approach eliminates the need for data registration with inspiratory CT scans. Results were analyzed with respect to the patients’ 3 years outcome to test their power to predict disease progression.

## Materials and methods

### Study design

This single-center retrospective longitudinal study consecutively included all patients diagnosed with IPF by a multidisciplinary interstitial lung disease (ILD) board between September 2016 and August 2019 who underwent CT scans in inspiration and expiration. All patients provided written informed consent, and the study was approved by the IRB (No. 10726_BO_K_2023). Other inclusion criteria were a pulmonary function test within +/- 50 days of the CT scan and at least one follow-up pulmonary function test (PFT) with a minimum delay of 6 months. None of the patients exhibited chronic comorbidities affecting the lungs, including pulmonary hypertension or emphysema. Patients suffering from acute pulmonary comorbidities at the time of the CT scan were also excluded. All of the study’s participants were treated with either pirfenidone or nintedanib.

### Image acquisition and processing

CT scans were obtained in supine position in full inspiration and maximal expiration using tube voltages of 150kV and 90 kV, respectively (mean computed tomography dose index (CTDI) 4.96 ± 1.59 mGy for inspiration, mean CTDI 3.70 ± 1.21 mGy for expiration, Somatom Force®, Siemens Healthineers). Patients were trained to cooperate with the breath command to the best of their abilities. Intravenous contrast agent (Iomeron 400, Bracco, Milano, Italy) was used in the scan acquisition protocol for perfusion assessment which was not relevant with respect to this study. Instead, virtual non-contrast images were generated using dedicated software (syngo.via® v 5.1, Siemens Healthineers). The acquisition protocol included tube voltages of 90 kV/ 150 kV, section intervals of 0.7 mm and a section thickness of 1.0 mm. Bolus tracking was performed in the left ventricle at a threshold of 200 HU and a delay of 12s for inspiration; the expiratory scan was performed with a 5 minute delay. Images were reconstructed using an advanced modeled iterative reconstruction algorithm (ADMIRE) and a soft Qr40 reconstruction kernel at a slice thickness of 1 mm.

Postprocessing included a lobe-wise segmentation of the lungs using an in-house automatic software based on a deep-learning algorithm [22]; manual corrections were made only when deemed necessary. All CT data were exported from our hospital’s picture archiving and communication system between 10/2/2023 and 24/2/2023.

### Clinical data

The PFTs with the shortest delay to the CT scan were used for clinical correlation, using a maximal time difference of 50 days as the inclusion criteria. To ensure better comparability between the patients, the follow-up PFT results were interpolated to exactly one-year after the respective baseline CT scan. This was achieved by deriving a line of best fit which considered factorial polynomial transformations up to the fourth degree. The differences between the baseline and one-year interpolated values were calculated and described as “delta”.

Patients were divided into two subgroups according to their status after their respective 3-year observation period. Endpoints were death or transplantation as measure for endstage pulmonary function failure for one group versus clinical surveillance for the other group. The threshold of three years was chosen based on the mean survival of patients with IPF, as reported in the literature [23,24]. All clinical data were extracted from the hospital information system on 18/2/2023.

### Statistical analysis

For each patient the following three histogram measures were calculated for inspiratory and expiratory CT scans separately:

- mean HU,
- skewness (measure of the asymmetry of the value distribution and is an indicator for the shift of the curve towards higher HU values in patients with fibrosis and increased collapse in expiration. Values for skewness decrease and may become negative),
- kurtosis (measure of the tailordness of the value distribution as compared to a normal distribution: a value < 3 refers to a broader (platykurtic) curve).

To quantify the extent of attenuation change during expiration as a function of the histogram in inspiration the following two measures were calculated:

- mean HU ratio (expiration [exp]/inspiration [insp]),
- the difference of the −250 HU percentile of the two histogram curves in inspiration and expiration (ΔP_(−250 HU)_). The threshold of −250 HU is used to separate the percentage of attenuation in the lungs above or below that value. Example: If a patient was on the 93^rd^ percentile in inspiration and on the 73^rd^ in expiration, the difference in the percentile is 20. The threshold of −250 HU was chosen as attenuation corresponding to alveolar collapse. Previous studies defined high attenuation areas ranging from −600 to −250 HU, which include ground-glass and reticular abnormalities but exclude denser areas like complete atelectasis, consolidation, blood vessels, and pulmonary nodules [25,26]. The threshold of −250 HU was thus at the lower end of parenchymal attenuation changes related to interstitial abnormalities.

All parameters were linearly correlated with the baseline forced vital capacity (FVC%) and the change in FVC% interpolated to one year follow-up (delta FVC%) using the Pearson correlation with a p-value of less than.05 for statistical significance after a two-tailed t-test.

A Mann-Whitney U test was used to compare all parameters between the two patient subgroups.

To evaluate the discriminative value of the measures, their impact was calculated by deriving three logistic regression models to predict death/transplantation after 3 years. Sensitivity analysis was also performed in a subset excluding patients who underwent transplantation.

- As the reference, a model which only included FVC% as a predictor was derived (FVC% model).
- A CT model was derived which included CT-derived histogram measures.
- Thirdly, a parsimonious model was calculated considering FVC% and all CT variables. For the parsimonious model, variables which contributed the least to the model were dropped from the model one by one until a model with the lowest Akaike information criterion was established. The area under the receiver operating characteristic curve (AUC) was calculated as an overall performance metric.

Additionally, all CT-derived histogram measures were assessed lobe-wise for both subgroups and presented graphically. To ascertain whether the observed variations in CT parameters between the upper lobes and lower lobes (RUL vs. RLL and LUL vs. LLL) were statistically significant, we employed paired t-tests.

Statistical analyses were performed by A.S. and S.C.S in statistical programs SPSS (version 25, IBM) and R (version 4.2.2).

## Results

### Study group

**Fig 1:**
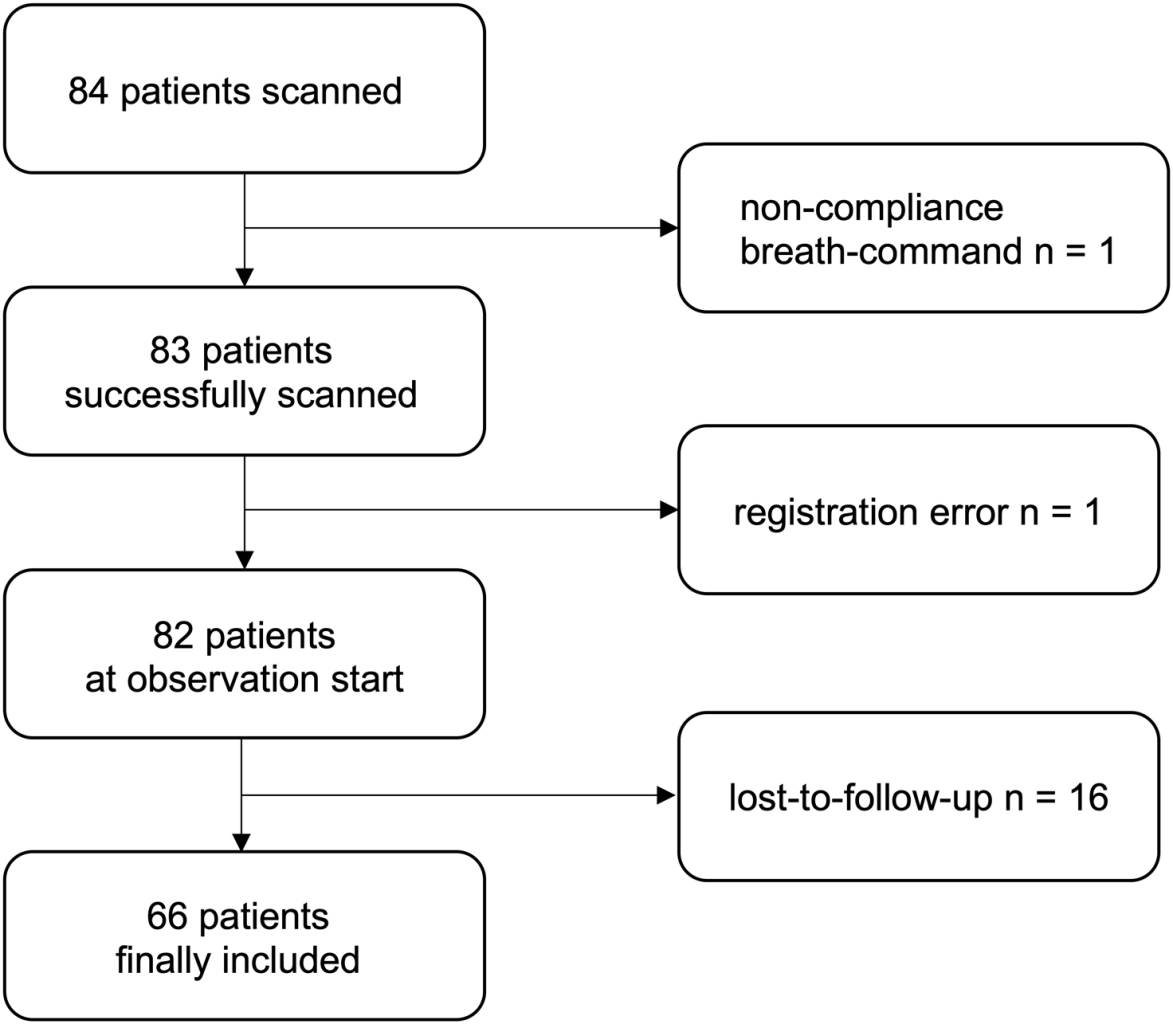
Flowchart for the different exclusion criteria.

The final study group consisted of 66 patients after exclusion of patients lost-to-follow-up, with lack of compliance during the scan or due to inaccurate registration, the latter only relevant for a comparative study we are currently working on (Fig 1).

Patient population characteristics are depicted in Table 1.

**Table 1:** Patient population characteristics.

No. of patients	66
**Sex**	
male	58 (88%)
female	8 (12%)
Age at baseline (years)	69.5 ± 9.7
**Smoking status**	
never	19 (29%)
former	41 (62%)
current	3 (5%)
unknown	3 (5%)
FVC% baseline	70.8 ± 16.9
FVC% interpolated to one yr.	66.8 ± 16.6
**Status after 3 years**	
death/transplantation	29 (44%)
in clinical surveillance	37 (66%)

Categorical variables are reported as frequencies with percent in brackets; continuous variables are reported as means ± standard deviations.

Most patients were males and were former smokers. After 3 years follow-up, 29 patients had either died or received a transplantation. Thirty-seven patients were still under clinical surveillance. FVC = forced vital capacity

The mean time interval between the CT scan and the baseline PFT was −4 ± 18 days.

In the death/transplantation group the mean time between the baseline CT and the respective event was 604 ± 294 days.

### Correlation between CT parameters and FVC%

Mean HU of the expiration showed a negative while skewness and kurtosis of the attenuation values in expiration showed a positive correlation with baseline FVC%.

Mean HU-ratio exp/ins showed no significant correlation. None of the expiratory histogram measures correlated with the delta FVC% (Table 2).

**Table 2:** Results of correlation analyses.

		Baseline FVC%	Delta FVC%
**ΔP**_**(−250 HU)**_	Pearson Correlation	−0.325^**^	0.104
	p (Sig. (2-tailed))	.008	.41
**mean HU ratio exp/insp**	Pearson Correlation	0.160	−0.008
	p (Sig. (2-tailed))	.20	.95
**mean-HU exp**	Pearson Correlation	−0.374^**^	0.084
	p (Sig. (2-tailed))	.002	.50
**skewness exp**	Pearson Correlation	0.409^**^	−0.111
	p (Sig. (2-tailed))	.001	.38
**kurtosis exp**	Pearson Correlation	0.358^**^	0.037
	p (Sig. (2-tailed))	.003	.77

Correlations between CT-derived histogram measures and baseline forced vital capacity (FVC%) baseline as well as delta FVC%. ΔP_(−250 HU)_ = difference in percentile values at −250 HU (between inspiration and expiration) in attenuation histograms

** = p-value <.01

### Differences between patient subgroups

All measures derived from CT scans showed significant differences between the two patient subgroups with skewness exp and ΔP_(−250 HU)_ achieving the smallest P value and the least overlap between mean and standard deviations (SD).

Delta FVC%, however, was not different between the subgroups (Table 3).

**Table 3:** Results of Mann-Whitney U test.

Parameter	In clinical surveillance (n = 37)		Death or transplantation (n = 29)		p*
	Mean	SD	Mean	SD	
**ΔP**_**(−250 HU)**_	8.09	5.88	13.9	8.02	<.001
**mean HU ratio exp/insp**	0.75	0.09	0.69	0.10	.008
**mean exp (HU)**	−575	94	−495	88	.002
**skewness exp**	0.99	0.46	0.55	0.37	<.001
**kurtosis exp**	3.62	1.25	2.67	0.48	.001
**baseline FVC%**	75.84	17.1	64.41	14.49	.005
**delta FVC%**	−4.43	6.62	−3.48	13.38	.75

Mann-Whitney U test for forced vital capacity (FVC%) and CT-derived histogram measures for the two subgroups. SD = standard deviation; ΔP_(−250 HU)_ = difference in percentile values at −250 HU (between inspiration and expiration) in attenuation histograms

### Logistic regression model

The three logistic regression models for predicting death or transplantation after 3 years follow-up are summarized in Table 4.

**Table 4:** Subgroup discriminatory performance with and without CT parameters.

	FVC% model		CT model		Parsimonious model	
Model parameter	Coefficient	Std. error	Coefficient	Std. error	Coefficient	Std. error
Intercept	2.925	1.231	−3.342	7.247	3.304	1.313
FVC%	−0.045	0.017	NA	NA	−0.026	0.018
ΔP_(−250 HU)_	NA	NA	−0.026	0.154	NS	NS
mean HU ratio exp/insp	NA	NA	0.530	9.560	NS	NS
mean exp	NA	NA	−0.017	0.016	NS	NS
skewness exp	NA	NA	−5.925	3.792	−2.280	0.799
kurtosis exp	NA	NA	−0.597	0.724	NS	NS
**Test characteristic**	**Value**	**95% CI**	**Value**	**95% CI**	**Value**	**95% CI**
AUC^1^	0.708	0.581-0.836	0.793	0.685-0.900	0.789	0.682-0.897

^1^95% CI calculated using DeLong method

FVC = forced vital capacity; NA = not applicable; NS = not significant (i.e., dropped from the parsimonious model); ΔP_(−250 HU)_ = difference in percentile Values at −250 HU (between inspiration and expiration) in attenuation histograms.

The overall discriminatory performance of the CT and the Parsimonious model performed similarly (AUC = 0.793, 95% confidence interval = 0.685–0.900 vs. 0.789, 0.682–0.897, respectively); both outperformed the FVC% model (0.708, 0.581–0.836), though the differences were not statistically significant because all AUCs were within each other’s confidence intervals.

The parsimonious model, which included only FVC% and skewness exp as predictors, was derived. Additional predictors such as ΔP_(−250 HU)_, mean HU ratio exp/insp, kurtosis exp, and mean exp did not provide additional predictive value.

### Lobe-wise values

**Fig 2:**
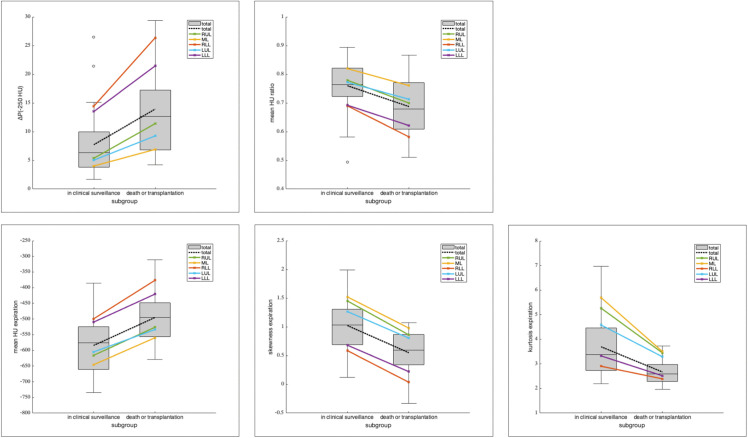
Regional differences in attenuation histogram metrics between inspiration and expiration. Difference in percentile values at −250 HU (between inspiration and expiration) in attenuation histograms (ΔP_(−250 HU)_) **(a)**, mean HU ratio exp/insp (b) and mean **(c)**, skewness (d) and kurtosis (e) of attenuation histograms in expiration in the subgroups are depicted as box charts for the total lung. Superimposed with color-coded lines are the mean values of the measure per total lung and per lobe for the two subgroups of patients. The lower lobes present with the highest mean density in expiration as well as with the smallest values for skewness, kurtosis and mean-HU-ratio compared to the upper lobes and the middle lobe. Differences between the two subgroups are most prominent in the lower lobes, and most striking for skewness exp and ΔP_(−250 HU)_.

Table 5 summarizes the mean and SD of the lobe-wise (RUL = right upper lobe; ML = middle lobe; RLL = right lower lobe; LUL = left upper lobe; LLL = left lower lobe) CT-derived histogram measures for the two subgroups. Fig 2 shows box charts of the results for the total lung, with mean values for each lobe superimposed. Paired t-test between the upper lobes and lower lobes (RUL vs. RLL and LUL vs. LLL) yielded highly significant results for all parameters and both sides of the lung, with p < 0.001 in each case.

**Table 5:** Lobe-wise values.

Parameter	Lobe	RUL	ML	RLL	LUL	LLL
ΔP_(−250 HU)_	In clinical follow-up (n = 37)	5.60 ± 4.74	4.01 ± 4.07	14.74 ± 10.39	5.60 ± 5.00	13.57 ± 12.06
	Death or transplantation (n = 29)	11.41 ± 8.55	6.91 ± 4.33	26.36 ± 12.48	9.25 ± 6.78	21.46 ± 13.20
mean HU ratio exp/insp	In clinical follow-up (n = 37)	0.77 ± 0.09	0.81 ± 0.07	0.68 ± 0.11	0.77 ± 0.09	0.69 ± 0.13
	Death or transplantation (n = 29)	0.70 ± 0.11	0.76 ± 0.09	0.58 ± 0.12	0.71 ± 0.10	0.62 ± 0.12
mean exp (HU)	In clinical follow-up (n = 37)	−607 ± 94	−641 ± 94	−491 ± 114	−595 ± 98	−502 ± 132
	Death or transplantation (n = 29)	−526 ± 104	−560 ± 72	−376 ± 88	−534 ± 97	−420 ± 108
skewness exp	In clinical follow-up (n = 37)	1.41 ± 0.54	1.52 ± 0.63	0.57 ± 0.54	1.23 ± 0.53	0.66 ± 0.61
	Death or transplantation (n = 29)	0.86 ± 0.55	0.98 ± 0.34	0.04 ± 0.37	0.80 ± 0.44	0.22 ± 0.45
kurtosis exp	In clinical follow-up (n = 37)	5.05 ± 1.89	5.65 ± 2.21	2.87 ± 0.80	4.48 ± 1.81	3.24 ± 1.27
	Death or transplantation (n = 29)	3.44 ± 1.11	3.50 ± 0.93	2.39 ± 0.46	3.29 ± 0.81	2.51 ± 0.55

Lobe-wise mean values ± SD of ΔP_(−250 HU)_, mean HU ratio exp/insp and mean, skewness and kurtosis of the expiratory attenuation histograms in both subgroups. LLL = left lower lobe; LUL = left upper lobe; ML = middle lobe; RLL = right lower lobe; RUL = right upper lobe; ΔP_(−250 HU)_ = difference in percentile values at −250 HU (between inspiration and expiration) in attenuation histograms.

### Case examples

**Fig 3:**
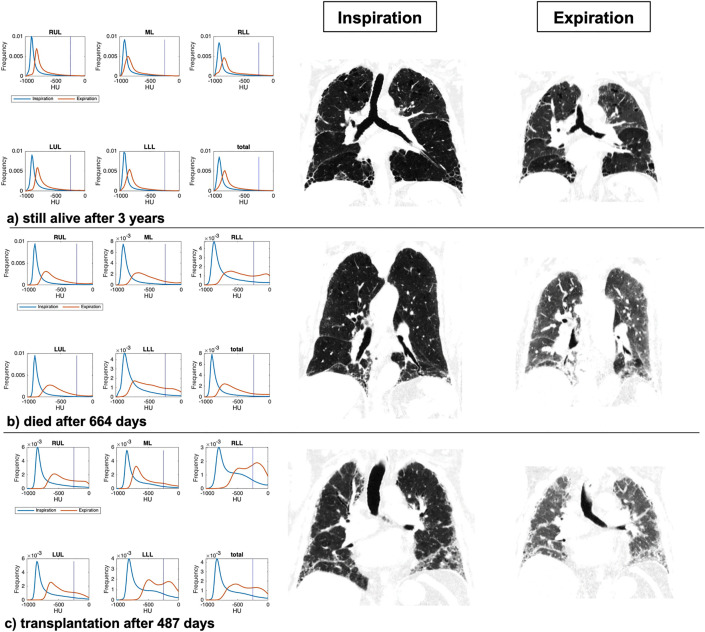
Lobe-wise and total lung attenuation histograms with corresponding CT images in inspiration and expiration. Three example patients (a, b, c) with their lobe-wise and total lung attenuation histograms (left) and coronal CT images in inspiration and expiration (right). The histograms display attenuation values on the x-axes (in HU) and frequency on the y-axis.

Three case examples are shown in Fig 3.

Patient a had only subtle fibrotic changes and remained under clinical surveillance throughout the 3-year observation period. The attenuation histograms demonstrate a typical distribution of attenuation values for normal lung parenchyma in inspiration.

In expiration the curve is slightly shifted to the right due to a moderate attenuation increase (skewness exp = 1.77, ΔP_(−250 HU)_ = 1.92). The FVC% was 89% at baseline and 85% at one-year follow-up.

Patient b died 664 days after the CT scan. The attenuation values in inspiration are slightly shifted towards higher attenuation.

In expiration, the attenuation curve is strongly shifted to the right towards higher attenuation values as indicated by a skewness exp of 0.85 and a ΔP_(−250 HU)_ of 11.04. Additionally, note the broadening of the expiratory histogram curve in the lower lobes (skewness exp RLL = 0.21, skewness exp LLL = 0.38). The FVC% was 88% at baseline and 83% at one-year follow-up.

Patient c underwent transplantation 487 days after the baseline CT scan. Similar to patient b, the curve in inspiration is already shifted towards higher attenuation values, especially in the lower lobes.

In expiration the curves are even more prominently shifted towards the right (skewness exp = 0.15 and ΔP_(−250 HU)_ = 22.62) with broadening of the curves and a double-peak in the lower lobes (skewness RLL = −0.15, skewness LLL = 0.06). The FVC% at baseline was 57% and 65% at one-year follow-up.

## Discussion

This study aimed to explore the value of attenuation histograms and their derived measures to depict differences in attenuation changes in paired inspiration/expiration scans in patients with IPF. Abnormally large attenuation changes are considered as surrogate for alveolar collapse in expiration, which has been described as precursor for development of fibrosis in IPF. Histopathological studies have demonstrated that a disrupted protein composition results in increased surface tension, which leads to alveolar collapse. While this is initially reversible, prolongation leads to piling of alveolar walls that eventually becomes irreversible and fibrotic [14,15].

Previous studies have shown that patients with IPF exhibit an abnormally high attenuation increase in expiration [16–19]. These studies, however, did not correlate this finding with patient outcome to find out whether it could be used as imaging biomarker to prognose progression of fibrosis. We therefore correlated CT-derived histogram measures in expiration and paired inspiration/expiration with future PFT impairment (one year follow-up) and 3-year patient outcome (survival/transplantation versus clinical follow-up). The objective was to assess the predictive value of abnormally high increased attenuation in expiration as a surrogate for alveolar collapse and subsequent progression of fibrosis in patients with IPF.

We found significant differences for FVC baseline as well as all CT-derived histogram measures. The subgroups showed the largest difference in skewness and ΔP_(−250 HU)_ in the Mann-Whitney U test. Skewness and mean attenuation in expiration were better predictors of death or transplantation at 3 years’ follow-up compared to the other CT measures.

The change in percentile values at −250 HU between inspiration and expiration corresponds to the relative change in lung volume during breathing above the −250 HU threshold. This measure seems therefore intuitive and readily comprehensible. While visual analysis favors the difference of the −250 HU percentile, statistics did not prove a significant improvement as compared to skewness in expiration.

The wide confidence intervals indicate that the findings require additional validation in a larger sample size. The threshold of −250 HU was chosen to approximate the attenuation of alveolar collapse, nevertheless more studies are needed to determine the optimal threshold. Alternatively, future studies could consider approaches integrating the full histogram shift, which may capture collapse-related density changes more comprehensively. Combining death and transplantation into a single outcome of disease progression may have also influenced the results. However, we followed the clinical reasoning from previous publications and considered both death and transplantation as indicators of end-stage respiratory failure [27,28]. Regardless, deriving prediction models in a subset excluding patients who underwent transplantation showed results which were equivalent to the primary prediction models (Table in S1 File).

Despite being considered a common and clinically widely applied measure for disease progression, it was found that delta FVC% was not different amongst the subgroups for endpoints.

The logistic regression analysis indicated that PFT may not be of added value for predicting death or transplantation within 3 years when quantitative CT measures are available.

Mean HU in expiration correlated negatively with FVC% baseline. Both, a high FVC% baseline value and a low mean attenuation are both indicative of normal lung parenchyma. Skewness and kurtosis showed a positive correlation with FVC% baseline. Lungs with more healthy parenchyma show histogram curves in inspiration and expiration that are more right-skewed and leptokurtic and exhibit large overlaps of the areas under the curves. Diseased lungs show a right shift of the attenuation values in inspiration but also a larger difference of the areas under the curves. The latter is an indicator for pathological behavior in expiration (alveolar collapse) and appeared to be correlated to bad prognosis according to our results. Additionally, the collapse is most prominent in the lower lobes, which is consistent with the dominant manifestation of IPF in that region. This finding indicates that these parameters accurately reflect the clinical picture.

Our results are in agreement with the findings previously published by Sul et al [17]. They also analyzed attenuation histograms of inspiratory and expiratory CT scans (in the publication described as contracted and non-contracted state) and observed a broadening of the curve and shift towards the right in patients with IPF. For all histogram derived measures, they found statistically significant differences between healthy (n = 13) and IPF patients (n = 9) and – similarly as in our results – these differences were larger in the contracted (expiratory) state. In that publication, however, no correlation with outcome was made. Moreover, the study groups were very small, and they did not consider measures taking both, inspiratory and expiratory information into account.

The present findings are also consistent with previous studies showing that regions that appear morphologically normal on inspiratory CT scans may nevertheless exhibit abnormal ventilation mechanisms, manifested by increased expiratory density or regional collapse, subsequently leading to fibrotic changes [19]. By incorporating expiratory imaging, this study highlights functional abnormalities that cannot be detected by morphological examinations alone. These ventilation-dependent density shifts can only be detected by paired inspiratory-expiratory analysis and thus complement existing quantitative CT approaches. Methods such as CALIPER or densitometry primarily characterize structural abnormalities that are already visible on inspiratory scans. In contrast, the expiratory collapse signal evaluated in this study could represent an earlier, dynamic indicator of impaired lung mechanics. As such, it could help identify “at-risk tissue” and offer potential value for assessing disease progression.

Our study has limitations, including a small sample size. To verify the results, further evaluation with a larger population from multiple institutions is needed. We also used virtual non-contrast images because the CT scans were performed with administration of contrast agent which were not relevant for this study. As this applied to all patients, we do not think it had a significant impact on the outcomes. Moreover, several studies showed a high comparability of VNC images and non-contrast images in other organs. Jungblut et al conducted a study in which VNC images were used to quantify emphysema. Their results showed no significant difference in emphysema quantification as compared to TNC [29]. Another potential confounder is the expiratory effort of the patient. The breath command for the scan is similar to the acquisition of PFT, therefore the patients are well trained. Certain inaccuracies remain nonetheless. Future studies could incorporate spirometric gating or volumetric correction to better control for variable respiratory effort.

Additionally, the retrospective nature of the cohort required interpolation of FVC values in patients with irregular follow-up intervals, which introduces additional imprecision and may obscure associations in the correlation analysis. Finally, the threshold of −250 HU was chosen to approximate the attenuation of alveolar collapse in expiration and fibrotic changes in inspiration. More studies are required to refine the optimal threshold.

In general, multicenter validation across scanners and protocols, integration of expiratory CT metrics into multidimensional prognostic models alongside established clinical and physiological variables, and evaluation of whether this functional marker can refine patient stratification — for example, identifying individuals who may benefit from earlier antifibrotic escalation or expedited referral for transplant assessment — is needed.

### Conclusion

The exploratory analysis of attenuation histogram-derived parameters in expiration and attenuation changes between inspiration and expiration might indicate alveolar collapse and could potentially serve as new prognostic imaging markers in idiopathic pulmonary fibrosis. This study may be regarded as a preliminary feasibility study; further research is necessary to ascertain its viability.

## Supporting information

S1 File Sensitivity analysis: Subgroup discriminatory performance with and without CT parameters, excluding patients who underwent a transplant. Sensitivity analysis on a dataset which includes 36 surviving patients (61%) without transplantation and 23 patients who died (39%). ^1^ 95% CI calculated using DeLong method; NA = not applicable; NS = not significant (i.e., dropped from the parsimonious model); FVC = forced vital capacity.
(DOCX)

## Abbreviations

FVC: forced vital capacity
HU: hounsfield units
IPF: idiopathic pulmonary fibrosis
LLL: left lower lobe
LUL: left upper lobe
ML: middle lobe
PFT: pulmonary function test
RLL: right lower lobe
RUL: right upper lobe
ΔP_(−250 HU)_: difference in percentile values at −250 HU (between inspiration and expiration) in attenuation histograms
