# Supplementary material for: Extent of alveolar collapse in expiratory CT as a prognostic marker in idiopathic pulmonary fibrosis
Source: PLoS One. 2026 Mar 17;21(3):e0345308. doi: 10.1371/journal.pone.0345308 (PMC12994813; doi:10.1371/journal.pone.0345308)
Supplement: S1 File — Sensitivity analysis on a dataset which includes 36 surviving patients (61%) without transplantation and 23 patients who died (39%). 1 95% CI calculated using DeLong method; NA = not applicable; NS = not significant (i.e., dropped from the parsimonious model); FVC = forced vital capacity. (DOCX) [file pone.0345308.s001.docx]

|  | FVC% model | | CT model | | Parsimonious model | |
| --- | --- | --- | --- | --- | --- | --- |
| **Model parameter** | **Coefficient** | **Std. error** | **Coefficient** | **Std. error** | **Coefficient** | **Std. error** |
| Intercept | 2.14172 | 1.28092 | -0.07555 | 8.21340 | 2.64119 | 1.34856 |
| FVC% | -0.03669 | 0.01794 | NA | NA | -0.01793 | 0.01926 |
| ΔP_(-250 HU)_ | NA | NA | 0.17497 | 0.17497 | NS | NS |
| mean HU ratio exp/insp | NA | NA | -2.12039 | 10.05160 | NS | NS |
| mean exp | NA | NA | -3.44705 | 4.36741 | NS | NS |
| skewness exp | NA | NA | -1.48101 | 1.09317 | -2.17779 | 0.92824 |
| kurtosis exp | NA | NA | -0.01655 | 0.01656 | NS | NS |
|  |  |  |  |  |  |  |
| **Test characteristic** | **Value** | **95% CI** | **Value** | **95% CI** | **Value** | **95% CI** |
| AUC^1^ | 0.681 | 0.541-0.822 | 0.769 | 0.650-0.889 | 0.746 | 0.621-0.872 |

**S1 Table. Sensitivity analysis: Subgroup discriminatory performance with and without CT parameters, excluding patients who underwent a transplant**

Sensitivity analysis on a dataset which includes 36 surviving patients (61%) without transplantation and 23 patients who died (39%).

^1^ 95% CI calculated using DeLong method

NA = not applicable; NS = not significant (i.e., dropped from the parsimonious model); FVC = forced vital capacity
